# Ischemic Preconditioning Does Not Alter Performance in Multidirectional High-Intensity Intermittent Exercise

**DOI:** 10.3389/fphys.2017.01029

**Published:** 2017-12-12

**Authors:** Christoph Zinner, Dennis-Peter Born, Billy Sperlich

**Affiliations:** ^1^Department of Sport Science, Julius-Maximilians-University Würzburg, Würzburg, Germany; ^2^Department of Sport, University of Applied Sciences for Police and Administration of Hesse, Wiesbaden, Germany; ^3^Department for Elite Sport, Swiss Federal Institute of Sport, Magglingen, Switzerland

**Keywords:** agility, change of direction, muscle oxygen saturation, near-infrared spectroscopy, team sport

## Abstract

**Purpose:** Research dealing with ischemic preconditioning (IPC) has primarily focused on variables associated to endurance performance with little research about the acute responses of IPC on repeated multidirectional running sprint performance. Here we aimed to investigate the effects of IPC of the arms and the legs on repeated running sprint performance with changes-of-direction (COD) movements.

**Methods:** Thirteen moderately-to-well-trained team-sport athletes (7 males; 6 females; age: 24 ± 2 years, size: 175 ± 8 cm, body mass: 67.9 ± 8.1 kg) performed 16 × 30 m all-out sprints (15 s rest) with multidirectional COD movements on a Speedcourt with IPC (3 × 5 min) of the legs (IPC_leg_; 240 mm Hg) or of the arms (remote IPC: IPC_remote_; 180–190 mm Hg) 45 min before the sprints and a control trial (CON; 20 mm Hg).

**Results:** The mean (±*SD*) time for the 16 × 30 m multidirectional COD sprints was similar between IPC_leg_ (Mean *t*: 16.0 ± 1.8 s), IPC_remote_ (16.2 ± 1.7 s), and CON (16.0 ± 1.6 s; *p* = 0.50). No statistical differences in oxygen uptake (mean difference: 0%), heart rate (1.1%) nor muscle oxygen saturation of the *vastus lateralis* (4.7%) and *biceps brachii* (7.8%) between the three conditions were evident (all *p* > 0.05).

**Conclusions:** IPC (3 × 5 min) of the legs (220 mm Hg) or arms (180–190 mm Hg; remote IPC) applied 45 min before 16 × 30 m repeated multidirectional running sprint exercise does not improve sprint performance, oxygen uptake, heart rate nor muscle oxygen saturation of the *vastus lateralis* muscle when compared to a control trial.

## Introduction

Repeated intervals of complete muscle blood flow restriction (2–4 intervals, duration 3–5 min) inducing ischemia, also known as ischemic preconditioning (IPC), has received clinical and practical interests, e.g., to protect the myocardium against a subsequent ischemic incident (Murry et al., 1986). After IPC application to the canine limbs the infarct area decreased by ~75% (Murry et al., 1986) with yet unclear mechanisms connected to phosphocreatine kinase induced cardio-protection in patients (Nouraei et al., 2016) and up-regulation of myocardial protein kinase C (Weinbrenner et al., 2002), circulation nitrites (Singh and Chopra, 2004) as well as calcitonin gene-related peptide in rats (Wolfrum et al., 2005). Later, the positive impact of IPC of the limbs was confirmed for internal organs and also for skeletal muscle (Gurke et al., 1996).

Latest exercise-related IPC research has primarily focused on variables associated to endurance performance. Acute improvements in peak oxygen uptake (+3%) (de Groot et al., 2010), power output at peak oxygen uptake (+4%) (Crisafulli et al., 2011), running time trial performance (+2.5%) (Bailey et al., 2012), 1,000-m rowing performance (+0.4%) (Kjeld et al., 2014), and time to task failure (+11.2%) (Barbosa et al., 2015) with IPC compared to a non-IPC trials have been documented. In this context a recent meta-analysis (Salvador et al., 2015) revealed that three intervals of 5-min IPC (220–250 mm Hg) performed 40–50 min before an exercise task are most promising to induce the aforementioned acute ergogenic responses of IPC vs. non-IPC trial. Earlier onset of exercise after IPC (<40–50 min) might results in decreased performance (Salvador et al., 2015) due to acute metabolic alterations induced by IPC such as decreased phosphocreatine levels, ATP and total adenosine nucleotides content as wells as higher blood carbon dioxide (CO_2_) levels immediately after IPC (Pang et al., 1995).

Interestingly, when IPC is applied to the limbs not involved in the subsequent exercise task (so-called “remote IPC”) the exercising muscles benefit from IPC due to systemic responses (Kjeld et al., 2014; Barbosa et al., 2015) related to circulating endogenous opioid peptides (Swyers et al., 2014) and endogenous substances activating ATP-sensitive potassium channels (Loukogeorgakis et al., 2007) without detrimental muscle metabolic alterations.

Recent studies have documented ergogenic effects of IPC in connection with repeated-sprint cycling (Patterson et al., 2015), land-based sprinting in team-sport athletes (Gibson et al., 2013), swimming (Jean-St-Michel et al., 2011), ergometer rowing (Kjeld et al., 2014), and handgrip exercise (Barbosa et al., 2015). The main ergogenic mechanisms have been attributed to enhanced phosphocreatine (PCr) resynthesis after ischemia (Lukes et al., 2005; Andreas et al., 2011), adenosine mediated vasodilation with accompanied enhanced oxygen (O_2_) delivery and ATP sparing (Liu et al., 1991; Jennings et al., 2001).

Relatively little research has focused on the acute responses of IPC on repeated high-intensity intermittent exercise performance with at this point of time inconclusive results. Recently, IPC of the legs (4 × 5 min IPC 45 min before sprint cycling) improved peak and mean power output significantly during the early stages (sprints 1, 2, 3) of repeated-sprint cycling (12 × 6 s, 5 min recovery) and could therefore reflect a promising IPC protocol for disciplines to improve (repeated) sprint performance (Patterson et al., 2015). In contrast, IPC of the legs (3 × 5 min IPC immediately before sprint cycling) before 5 × 6 s maximal cycle sprint efforts (with 24 s recovery) revealed no performance benefit when compared to a non-IPC control trial (Gibson et al., 2015). Even though the influence of IPC on repeated high-intensity intermittent exercise performance is inconclusive, team sports may benefit from IPC since (i) repeated high-intensity intermittent exercise is an important independent variable for team sports (Taylor et al., 2016) and (ii) involves aerobic components as well as a rapid ATP production via phosphocreatine resynthesis (Bishop et al., 2011). In addition, most high intensity exercise in team sports involve change-of-direction (COD) movements increasing the neuro-muscular demand even further (Bloomfield et al., 2007). Since IPC of the legs and arms has shown to improve intermittent high-intensity cycling performance and associated physiological variables [e.g., peak oxygen uptake (de Groot et al., 2010), oxygen uptake kinetics (Paganelli et al., 1989), muscle oxygenation (Saito et al., 2004)], the question remains whether IPC of the legs or arms could be a simple, socially acceptable and inexpensive ergogenic strategy to enhance multi directional repeated high-intensity intermittent running.

Therefore, the aim of the present investigation was to test the hypothesis that IPC of the legs or arms could improve the repeated high-intensity intermittent exercise involving COD movements in moderately-to-well-trained team-sport athletes.

## Materials and methods

### Participants

Thirteen moderately-to-well-trained team-sport athletes (football, basketball, handball; 7 males; 6 females; age: 24 ± 2 years, size: 175 ± 8 cm, body mass: 67.9 ± 8.1 kg) with experience in team sports gave their written informed consent to participate in this study, which was approved by the ethics review board of the Institute of Sport Science of the University of Würzburg in accordance with the 1964 Helsinki declaration and its later amendments. Prior to testing, the participants were fully familiarized with the laboratory exercise procedures. On the test days, all were asked to report to the laboratory well-hydrated, having consumed a light meal at least 2 h earlier, and not having performed any strenuous exercise during at least 24 h before the tests.

### Procedures

All participants reported to the laboratory four times. During the initial visit, anthropometric data was obtained and the participants were familiarized with the 16 × 30 m (i.e., ~15 s effort duration) all-out multidirectional changed of direction sprints followed by 15 s recovery. The second, third and fourth visit consisted of either ischemic preconditioning of the legs (IPC_leg_), of the arms (remote IPC: IPC_remote_) or a control trial (CON) performed 45 min before the multidirectional repeated sprints. IPC_leg_, IPC_remote_, CON were performed in a counterbalanced order, with 5–7 days in-between to eliminate any possible carryover effects between the trials (Loukogeorgakis et al., 2005). All testing sessions took place at the same time of the day between 10:00 a.m. and 2:00 p.m. For all testing the participants wore the same shoes and clothes. They were asked to consume a cereal bar (55 g energy bar, PowerBar), water and a banana.

Noteworthy, we did not further assess the phase of the menstrual cycle of the females, therefore we cannot judge the potential of menstrual cycle as a confounding factor.

### Measures

#### Ischemic preconditioning protocol

The IPC protocol was performed in a supine position using bilateral occlusion of the legs (IPC_leg_) or arms (IPC_remote_), respectively (de Groot et al., 2010; Bailey et al., 2012). An automatic occluding cuff (14.5-cm width; Moor Instruments, Devon, UK) was positioned proximally around the upper thighs or arms and inflated for 5 min to 240 mm Hg during IPC_leg_ and 5 min to 180–190 mm Hg during IPC_remote_ followed by 5 min of reperfusion (Marocolo et al., 2016). This procedure was repeated three times as recommended elsewhere (Salvador et al., 2015). CON was identical to IPC_leg_ except that the cuffs were inflated to 20 mm Hg as performed elsewhere (Patterson et al., 2015). The warm-up for the sprints started 40 min after the removal of the cuffs as IPC has been shown to improve exercise performance 40–50 min after termination of IPC (Salvador et al., 2015).

#### Sprint protocol

Forty minutes after the IPC protocol the participants warmed-up for 5 min at a self-selected moderate exercise intensity (corresponding to 13 “somewhat hard” on Borg's 6–20 scale; Borg, 1970) involving multidirectional COD movements on the Speedcourt (Globalspeed GmbH, Hemsbach, Germany) which is a platform with the dimensions of 5 × 5 m and 12 contact plates integrated in a symmetric order. The specifications of the Speedcourt and its re-test reliability of the multidirectional and repeated sprints have been described in detail previously (Duking et al., 2016). Technical error of measurements of repeated-multidirectional sprint times on the Speedcourt was determined with an ICC of >0.79 and CV of <5%. After the warm-up procedure, the participants performed the 16 × 30 m repeated sprints with multidirectional COD movements.

During each multidirectional sprint sequence the players had to touch 16 contact plates with their foot as quickly as possible. The length of the running paths was 30 m which corresponded to ~15 s of multidirectional sprinting. On a visual countdown (displayed by the flat screen of the Speedcourt), the players had to sprint to the first contact plate and after foot touch-down the next contact plate was visualized to the athlete. After IPC_leg_, IPC_remote_, and CON 16 consecutive sprint intervals at a work-to-rest ratio of ~1:1 (~15:15 s) were performed.

The running paths of the repeated sprints for each 1st, 2nd, 3rd, and 4th of the 16 sprints were pre-defined. This sequence of these four intervals was repeated four times to analyze the performance for the beginning (Sprint 1–4), early midsection (Sprint 5–8), late midsection (Sprint 9–12), and end (Sprint 13–16) of the protocol. The exact same protocol was applied for all three conditions, the IPC_leg_, IPC_remote_, and CON. However, the running paths were unknown to the participants and none of the participants reported to have realized that the paths were repeated throughout the repeated sprints.

#### Cardiorespiratory measures

Oxygen uptake and heart rate (H7 heart rate belt, Polar Electro Oy, Kempele, Finland) were monitored continuously throughout the multidirectional repeated sprints with an open circuit breath-by-breath analyzer (Cortex, MetaMax 3B, Leipzig, Germany). The gas analyzer was calibrated prior to each test using a two-point calibration procedure encompassing the range of the anticipated fractional concentration of oxygen and carbon dioxide (room air containing 20.9% O_2_ and 0% CO_2_ as well as a gas mixture consisting of 15.8% O_2_ and 5% CO_2_ in N_2_; Praxair, Düsseldorf, Germany). The volume sensor was calibrated using a precision 3-L syringe (Cortex, Leipzig, Germany). The sampling line was replaced after two tests. The percentage errors and percentage technical error of measurements of oxygen uptake in repeated measures with Metamax 3B was determined to be <2% (MacFarlane and Wong, 2012).

#### Near-infrared spectroscopy

During the IPC as well as all multidirectional repeated sprints, the oxygenation of the *m. vastus lateralis* and of the lateral portion of the *m. biceps brachii* was measured by near-infrared spectroscopy with a sampling frequency of 1 Hz (NIRS; Moxy Monitor, Fortiori Design LLC, Spicer, MN, USA). Alterations of oxygenated and deoxygenated hemoglobin allow the calculation of the percentage of hemoglobin containing O_2_ (SmO_2_). Assuming that the light passing into micro-vessels which is >1 mm is completely absorbed, the major part of the reflected light originates from capillaries reflecting the relative supply and uptake of O_2_ by the muscle (McCully and Hamaoka, 2000; FortioriDesign LLC, 2015). The lowest single value (with 1 Hz sampling) of the SmO_2_ of every sprint for the *m. vastus lateralis* and the *m. biceps brachii* was applied for statistical analysis. For the data of the IPC protocol the mean SmO_2_ of the 3 × 5 min IPC maneuver and recovery periods were used for statistical analysis. Previously, the Moxy showed strong correlations between SmO_2_ values during two repeated cycling exercise protocols, conducted 1 week apart (Crum et al., 2017). Furthermore, our own preliminary data derived from repeated multidirectional sprint tests showed a TEM of minimum (de-oxygenation) and maximum (re-oxygenation) SmO_2_ to be 1.8 and 2.3%, respectively.

#### Statistical analysis

Calculations by conventional procedures provided the mean values and standard deviations (*SD*) for all sets of data. Testing revealed that all of the data were normally distributed without any further transformation necessary. Analyses of variance (ANOVA) were performed to seek for differences in the parameters under the three different interventions (main effects of trial and sprint block). In the case of global significance Tukey *post-hoc* analysis identified differences between the time-points. An alpha of *p* < 0.05 was considered to be significant and all analyses were carried out with the Statistical software package for Windows® (version 7.1, StatSoft Inc., Tulsa, OK, USA). The effect size Cohen's d (defined as the difference between the means divided by the standard deviation; Cohen, 1988) was calculated and the thresholds for small, moderate, and large effects defined a priori as 0.20, 0.50, and 0.80, respectively (Cohen, 1988).

The smallest worthwhile effect was the smallest Cohen change in the mean: 0.2 times the between-subject *SD* for the mean time of the 16 × 30 m multidirectional sprints of all participants (Batterham and Hopkins, 2006). Chances of benefit or harm were assessed qualitatively as follows: <1% almost certainly none, 1–5% very unlikely, 5–25% unlikely, 25–75% possibly, 75–95% likely, 95–99% very likely, >99% almost certainly (Hopkins, 2002).

## Results

The mean time for the 16 × 30 m multidirectional sprints on the Speedcourt was similar between IPC_leg_, IPC_remote_, and CON (16.0 ± 1.8, 16.2 ± 1.7, 16.0 ± 1.6 s, *p* = 0.50, respectively). Furthermore, there was no difference between the three conditions when comparing the beginning, midsections and end of the 16 × 30 m protocol (all *p* > 0.98; Table 1). The fatigue index was not different between the groups and all time points (*p* > 0.77). The mean sprint time did not differ between time points nor between IPC_leg_, IPC_remote_, and placebo (*p* > 0.50; Table 2).

**Table 1:** Mean (±*SD*) sprint time, sprint times for the four segments, and cardio-respiratory parameters during the 16 × 30 m multidirectional sprints of the thirteen participants.

		**IPC_leg_**	**IPC_remote_**	**CON**
Mean time (s)	16 × 30 m	16.0 ± 1.8	16.2 ± 1.7	16.0 ± 1.6
	Sprint 1–4	15.8 ± 1.8	16.0 ± 1.8	16.0 ± 1.7
	Sprint 5–8	16.0 ± 1.9	16.2 ± 1.4	15.9 ± 1.5
	Sprint 9–12	16.3 ± 1.9	16.5 ± 1.9	16.2 ± 1.6
	Sprint 13–16	16.1 ± 1.7	16.2 ± 1.5	16.1 ± 1.5
Oxygen uptake (L/min)	16 × 30 m incl. recovery	2.9 ± 0.7	2.9 ± 0.6	2.9 ± 0.7
	Sprint 1–4	2.8 ± 0.7	2.8 ± 0.7	2.9 ± 0.7
	Sprint 5–8	3.1 ± 0.6	3.2 ± 0.7	3.2 ± 0.7
	Sprint 9–12	3.2 ± 0.7	3.2 ± 0.7	3.3 ± 0.7
	Sprint 13–16	3.2 ± 0.7	3.2 ± 0.7	3.3 ± 0.7
Respiratory exchange ratio (a.u.)	16 × 30 m incl. recovery	1.05 ± 0.06	1.06 ± 0.04	1.05 ± 0.07
	Sprint 1–4	1.02 ± 0.11	1.02 ± 0.10	1.01 ± 0.11
	Sprint 5–8	1.11 ± 0.11	1.11 ± 0.06	1.11 ± 0.11
	Sprint 9–12	1.06 ± 0.07	1.07 ± 0.05	1.08 ± 0.08
	Sprint 13–16	1.04 ± 0.06	1.04 ± 0.04	1.04 ± 0.06
Ventilation (L/min)	16 × 30 m incl. recovery	104 ± 27	105 ± 27	104 ± 29
	Sprint 1–4	93 ± 24	92 ± 24	94 ± 26
	Sprint 5–8	114 ± 26	112 ± 25	117 ± 31
	Sprint 9–12	117 ± 27	117 ± 28	122 ± 34
	Sprint 13–16	118 ± 28	120 ± 29	124 ± 34
Heart rate (bpm)	16 × 30 m incl. recovery	176 ± 9	178 ± 10	178 ± 10
	Sprint 1–4	167 ± 15	169 ± 13	168 ± 15
	Sprint 5–8	178 ± 9	180 ± 9	179 ± 10
	Sprint 9–12	181 ± 9	183 ± 9	183 ± 10
	Sprint 13–16	184 ± 10	186 ± 10	185 ± 10

**Table 2:** Effects of IPC_leg_ and IPC_remote_ class on 16 × 30 m multidirectional sprint performance.

**Comparison**	**16** × **30 m sprint**	
	**Mean effect^a^ ± 90% CI^b^**	**Qualitative inference**
IPC_leg_ vs. IPC_remote_	0.1 ± 0.5	Unclear
IPC_leg_ vs. CON	0.0 ± 0.4	Unclear
IPC_remote_ vs. CON	0.1 ± 0.4	Unclear

*Qualitative inference represents the likelihood that the true value will have the observed magnitude*.

a *Mean effect refers to the first group minus the second group*.

b *±90% CI, add and subtract this number to the mean effect to obtain the 90% confidence intervals for the true difference*.

All cardio-respiratory data during the repeated multidirectional sprints are shown in Table 1. No differences were evident between the three conditions for oxygen uptake (*p* = 0.99), respiratory exchange ratio (*p* = 0.92), ventilation (*p* = 0.99), and heart rate (*p* = 0.86) in total or for any of the beginning, early and late midsections, and end of the 16 × 30 m protocol (all *p* > 0.80).

The mean oxygenation during IPC, reperfusion, and exercise are shown in Table 3. During IPC the SmO_2_ declined significantly in the restricted limbs (*p* < 0.001) and during the 5 min of reperfusion the SmO_2_ increased back to normal levels (*p* = 0.92). During the sprints, no differences in the mean SmO_2_ of the *m. vastus lateralis* or *m. biceps brachii* between the conditions were evident (all *p* > 0.75). The lowest mean SmO_2_ as well as the re-oxygenation during the sprints did not differ between IPC_leg_, IPC_remote_, and placebo at any time point (all *p* > 0.64) (Table 4).

**Table 3:** SmO_2_ of the *m. vastus lateralis* and *m. biceps brachii* in % during IPC, reperfusion.

**Condition**	**Muscle**	**Time point**		
		**Baseline**	**IPC**	**Reperfusion**
IPC_leg_ (%)	Vastus lateralis	71.5 ± 7.4	38.2 ± 10.5	75.7 ± 5.9^a^
	Biceps brachii (no IPC)	60.8 ± 7.7	62.9 ± 7.5	
IPC_remote_ (%)	Vastus lateralis (no IPC)	70.6 ± 8.0	75.3 ± 8.9	
	Biceps brachii	66.3 ± 6.7	25.9 ± 5.4	65.5 ± 6.6^a^
CON (%)	Vastus lateralis (no IPC)	69.4 ± 10.6	74.6 ± 9.9	
	Biceps brachii (no IPC)	54.3 ± 5.0	59.9±, 7.5	

a *Significant difference to IPC*.

**Table 4:** Mean (±*SD*) SmO_2_ for the four segments during the 16 × 30 m multidirectional sprints of the thirteen participants for the *m. vastus lateralis* and the *m. biceps brachii*.

		**IPC_leg_**	**IPC_remote_**	**CON**	**IPC_leg_**	**IPC_remote_**	**CON**
		**Maximum re-oxygenation of SmO**_**2**_ **during recovery**			**Maximum de-oxygenation of SmO**_**2**_ **during sprints**		
*m. vastus lateralis* (%)	Sprint 1–4	73.9 ± 18.8	73.6 ± 18.8	70.5 ± 22.1	39.6 ± 12.2	35.2 ± 15.7	48.8 ± 23.5
	Sprint 5–8	73.2 ± 11.0	73.0 ± 12.0	68.9 ± 13.8	45.9 ± 12.8	41.0 ± 12.2	52.9 ± 23.4
	Sprint 9–12	70.2 ± 10.5	70.2 ± 14.7	71.4 ± 12.4	47.1 ± 13.1	41.5 ± 13.1	58.7 ± 22.4
	Sprint 13–16	69.1 ± 14.3	69.5 ± 14.0	72.5 ± 10.7	47.9 ± 14.3	46.0 ± 12.5	56.7 ± 22.1
*m. biceps brachii* (%)	Sprint 1–4	65.9 ± 27.9	67.3 ± 26.7	59.5 ± 28.5	45.3 ± 22.3	50.6 ± 18.8	50.3 ± 17.8
	Sprint 5–8	49.3 ± 22.2	50.8 ± 21.7	43.9 ± 19.6	40.1 ± 23.2	50.0 ± 19.2	43.7 ± 20.0
	Sprint 9–12	52.7 ± 16.7	58.8 ± 20.2	50.3 ± 16.3	45.5 ± 23.2	56.3 ± 19.5	45.1 ± 18.3
	Sprint 13–16	60.8 ± 15.6	62.8 ± 20.1	51.2 ± 14.5	53.0 ± 24.4	60.6 ± 21.9	49.0 ± 17.4

*Data is presented as % of the baseline value*.

## Discussion

The present study was designed to test the hypothesis that repeated multidirectional sprint performance can be enhanced or not by IPC_leg_ or IPC_remote_ in moderately-to-well-trained team sport athletes. The major findings were that neither IPC_leg_ nor IPC_remote_ applied 45-min before the repeated multidirectional sprints improve performance or any selected physiological parameter when compared to placebo trial.

Multidirectional repeated sprint performance is an important prerequisite for success in most team-sport events (Bloomfield et al., 2007). Since IPC before repeated-sprint cycling improved performance when compared to non-IPC repeated-sprint cycling (Patterson et al., 2015), we were curious if the ergogenic IPC response in repeated-sprint cycling could be transferred to multidirectional repeated sprinting as performed in most team sports. Based on our results IPC does not alter performance or any selected physiological parameter.

As pointed out earlier (Patterson et al., 2015), the increase in sprint performance after IPC may be a result of altered energy contribution including phosphocreatine re-synthesis after ischemia (Lukes et al., 2005; Andreas et al., 2011), adenosine mediated vasodilation with accompanied enhanced oxygen delivery and ATP sparing (Liu et al., 1991; Jennings et al., 2001). NIRS-based data has been utilized to investigate the level of oxygenation in human skeletal muscle which reflects the balance between oxygen supply and utilization (Hamaoka et al., 1996). In the present study, the SmO_2_ of the *vastus lateralis* and *biceps brachii* muscle was unaltered between IPC_leg_, IPC_remote_, and CON. At this point, we cannot judge whether oxygen supply and/or elevated oxygen utilization changed, but since the NIRS-based SmO_2_, systemic oxygen uptake as well as heart rate were unaltered, we may assume that neither oxygen supply nor oxygen utilization was different between IPC_leg_, IPC_remote_, and CON. In this context some studies reported a reduced IPC lactate accumulation after exercise. This might be due to enhanced mitochondrial function after IPC (Andreas et al., 2011).

In a recent study involving 12 × 6 s repeated-sprint cycling with 30 s of passive recovery 30 min after IPC, the peak power output during the first few sprints was significantly increased (Patterson et al., 2015) compared to the repeated-sprint cycling without IPC, potentially due to the aforementioned metabolic IPC-induced alterations. However, it is noteworthy that although the IPC protocol before repeated sprint cycling induced significant improvements in peak power output (+2–3%) compared to a repeated sprinting without IPC (Patterson et al., 2015), the difference was ~36 W which is within the technical error range for this type of ergometer (±5% for power >1,500 W according to the manufacturer). The improved power output of +2–3% in sprint cycling (Patterson et al., 2015) after IPC may therefore be of minor practical relevance.

Since (i) other (Gibson et al., 2015) and our results showed no difference in repeated-sprint performance between IPC_leg_, IPC_remote_, and CON and (ii) the effect of IPC before repeated-sprint cycling was small (and potentially practically irrelevant; Patterson et al., 2015) we may conclude that neither IPC_leg_ nor IPC_remote_ before repeated sprints may improve multidirectional repeated sprint performance with a worthwhile effect.

It has been suggested that the efficacy of IPC might be determined by the volume of tissue exposed to the stimulus (Loukogeorgakis et al., 2005) and that there is a threshold for the amount of ischemic stimuli needed to elicit enhancement of performance (Kraus et al., 2015). This is supported by recent IPC related research. Remote IPC means that the physiological effects of IPC do not directly affect the same part of system of the body where it is applied. It is suggested that the ischemic event, induced by remote IPC, potentially leads to the production of substances that reach other tissues in the body where protective effects are induced (Przyklenk et al., 1993).

In the present study neither IPC_remote_ or IPC_leg_ improve performance when compared to CON indicating (at least in our study when involving leg exercise) that the increase of volume of tissue exposed to the IPC stimulus did not show any “threshold effect.” The present results have also been confirmed earlier (Salvador et al., 2015). Potentially the simultaneous combination of IPC of the arms and legs could favor improved repeated-sprint performance which is subject for future investigation.

With regards to team sports, the practicability of IPC can be questioned. Applying IPC 40–50 min before the beginning of a match might be possible, even though we did not find any mean effects some athletes might use this procedure, but would dramatically alter the pre-match warm-up routines. So far, no study has investigated the sustainability of the potential ergogenic effects (Patterson et al., 2015) of IPC which might be to transient to last for a half-time of 45 min in soccer match play. Finally, the painful procedure of IPC might further question the acceptability by the athletes to apply this method during their pre-match warm-up routines despite the potential effects on multi-directional sprint performance.

## Conclusion

The present study aimed to test the hypothesis that repeated multidirectional sprint performance can be enhanced by IPC_leg_ or IPC_remote_ in moderately-to-well-trained team-sport athletes. We conclude that ischemic precondition (3 × 5 min) of the legs (240 mm Hg) or arms (180–190 mm Hg) 45 min before 16 × 30 m repeated multidirectional sprints does not improve sprint performance, oxygen uptake, heart rate nor muscle oxygen saturation of the *vastus lateralis* and *biceps brachii* muscle when compared to repeated sprinting without IPC.

## Author contributions

All authors listed have made a substantial, direct and intellectual contribution to the work, and approved it for publication.

### Conflict of interest statement

The authors declare that the research was conducted in the absence of any commercial or financial relationships that could be construed as a potential conflict of interest.
